# Supplementary material for: Sodium Butyrate Supplementation in Whole Milk Modulates the Gastrointestinal Microbiota Without Altering the Resistome and Virulome in Preweaned Calves
Source: Microorganisms. 2025 Oct 15;13(10):2375. doi: 10.3390/microorganisms13102375 (PMC12566572; doi:10.3390/microorganisms13102375)
Supplement: Supplementary file 1 [file microorganisms-13-02375-s001.zip › microorganisms-3871369-supplementary.pdf]

Supplementary Table S1. Ingredients and chemical composition of starter mixture and whole milk (on DM basis).

Composition	Item[1]	
	SM	WM
Ingredients, g/kg as–fed		
Corn	370	-
Wheat bran	155	-
Soybean meal	390	-
Oil bran	25	-
Salt	1.8	-
Sodium bicarbonate	8.2	-
Calves starter premix[2]	50	-
Sodium butyrate	-	-
Total	100	
Chemical composition, g/kg		
DM, %	91.71	12.6
CP, %	23.43	-
EE, %	14.75	-
NDF, %	22.47	-
ADF, %	7.08	-
Ash, %	10.68	-
Milk protein, %	-	3.21
Milk fat, %	-	3.96
Total solid, %	-	12.89
Lactose, %	-	4.73

[1]SM = starter mixture; WM = whole milk.

[2]The premix provided per kg of the starter was as follows: V_A 13,050 IU, V_D 3262 IU, V_E 260.997 IU, Fe 116.817 mg, Cu 19.621 mg, Mn 48.516 mg, Zn 74.603 mg, Se 0.746 mg, I 1.343 mg, Co 0.966 mg.

Supplementary Table S2. Information on the sequencing depth of 16S rRNA genes.

Sample ID	Total read bases (bp)	Clean reads	Mean_length	Min_length	Max_length	Good's-coverage
**Rumen**						
**CON1**	22237965	53354	416.800334	231	505	0.996321
**CON2**	19733760	47422	416.13091	224	470	0.996818
**CON3**	17569349	41596	422.380734	250	431	0.998508
**CON4**	20376320	49437	412.167405	284	493	0.996917
**CON5**	22154975	53453	414.475801	235	525	0.996519
**CON6**	21854153	52420	416.904865	259	485	0.996884
**SB1**	19905662	47335	420.527348	262	516	0.997713
**SB2**	16573109	39287	421.84715	402	430	0.998011
**SB3**	17096427	40374	423.451404	388	458	0.99874
**SB4**	24073018	57622	417.774774	205	469	0.996851
**SB5**	20455321	49240	415.420816	265	489	0.997381
**SB6**	18513129	44895	412.365052	336	510	0.998376
**Cecum**						
**CON1**	27512954	66979	410.769853	208	474	0.991083
**CON2**	17179556	41560	413.367565	259	432	0.99589
**CON3**	20915910	50751	412.128037	262	475	0.994829
**CON4**	24891438	60254	413.108474	228	532	0.993801
**CON5**	25033767	60407	414.418312	232	436	0.993337
**CON6**	22567532	54549	413.711195	235	462	0.994299
**SB1**	25314415	61253	413.276329	235	466	0.99231
**SB2**	21176275	51702	409.583285	232	469	0.99463
**SB3**	24369772	58856	414.057564	266	509	0.994199
**SB4**	25012863	60534	413.203539	236	467	0.992542
**SB5**	25624527	62510	409.926844	203	464	0.991978
**SB6**	22734430	55155	412.191642	261	441	0.994497
Summation		1260945				
Average	21786526.13	52539.375	414.9995513	258.25	475.3333333	0.995518

Supplementary Table S3. Ruminal and cecum microbial community alpha diversity indices

	CON	SB	SEM	*P*-value
Rumen				
Chao1	1899.83	1761.67	85.29	0.28
Shannon	3.89	3.79	0.14	0.59
Simpson	0.07	0.08	0.01	0.90
Cecum				
Chao1	2003.83	2007.50	74.20	0.97
Shannon	3.95	3.91	0.001	0.54
Simpson	0.04	0.05	0.002	0.67

The control group was the calves fed whole milk without SB supplementation (CON group) and the SB group fed whole milk with 8.8 g/day SB supplementation. n = 6 for each group

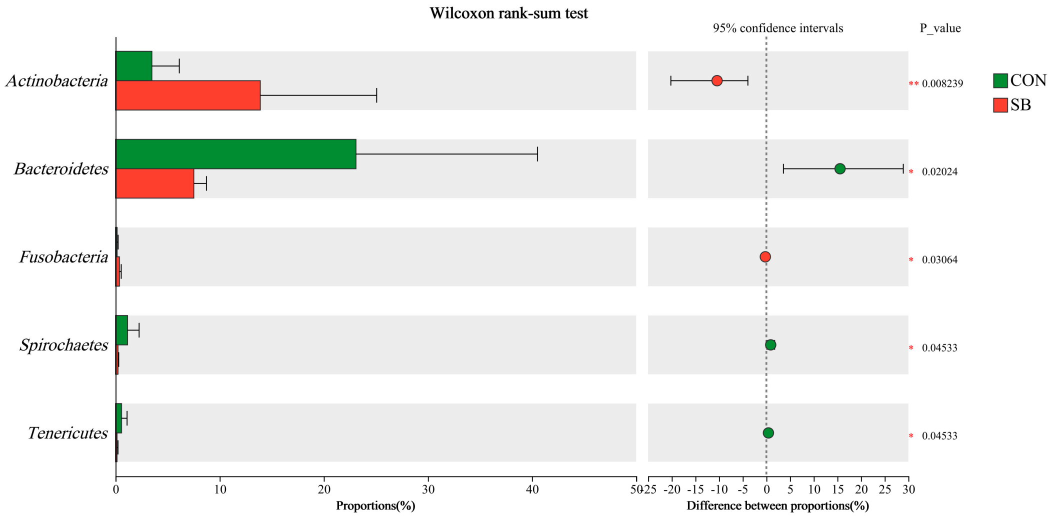

A

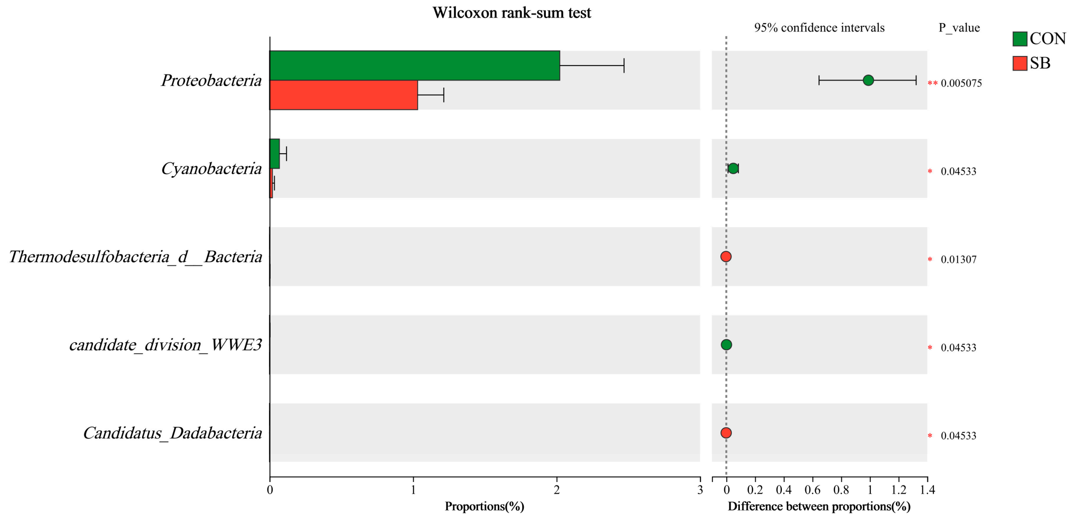

B

Supplementary Figure S1. Effects of sodium butyrate supplementation in milk on the taxonomic analysis of the rumen and cecum microbiota in preweaning calves at the phylum levels.

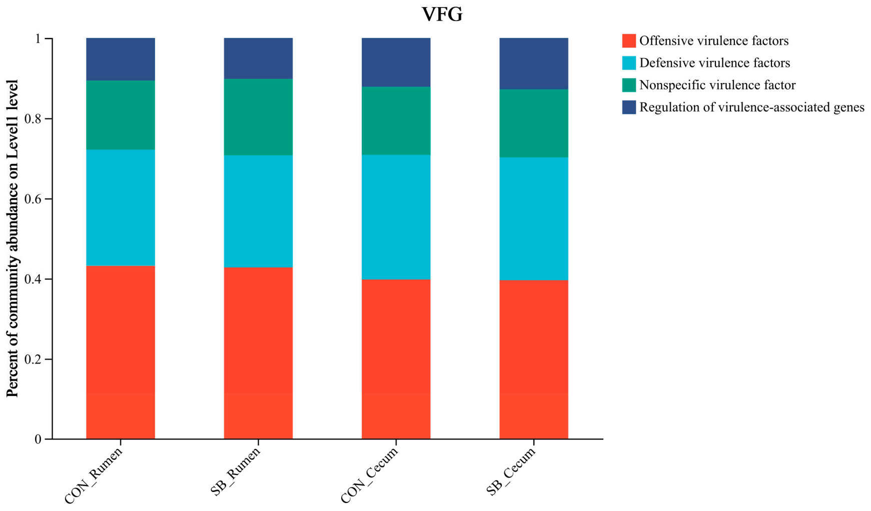

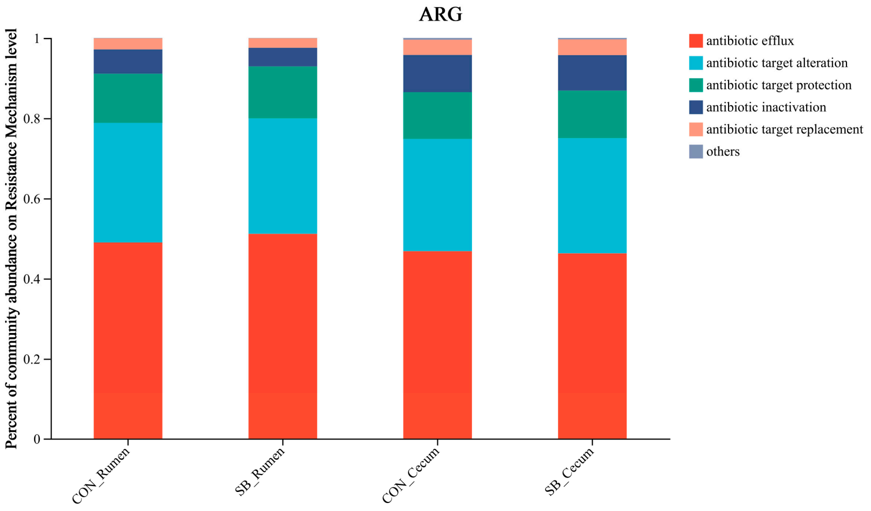

A

B

Supplementary Figure S2. Effect of sodium butyrate (SB) added to whole milk on rumen (A) and cecum (B) VFGs and ARGs in preweaned calves. The control group was the calves fed whole milk without SB supplementation (CON group) and the SB group fed whole milk with 8.8 g/day SB supplementation. n = 6 for each group

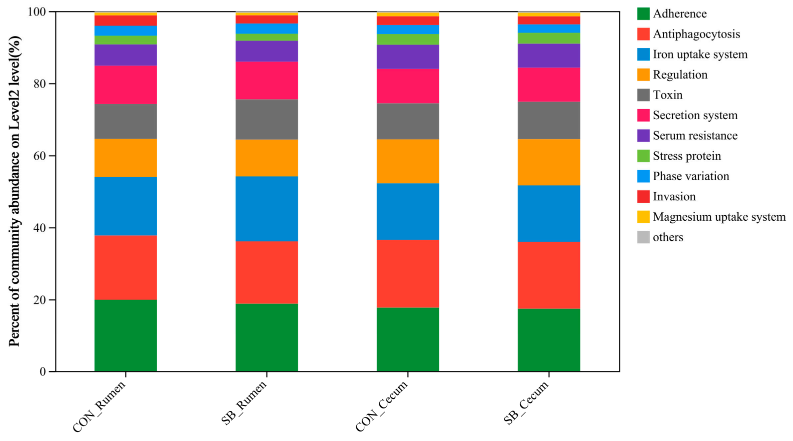

A

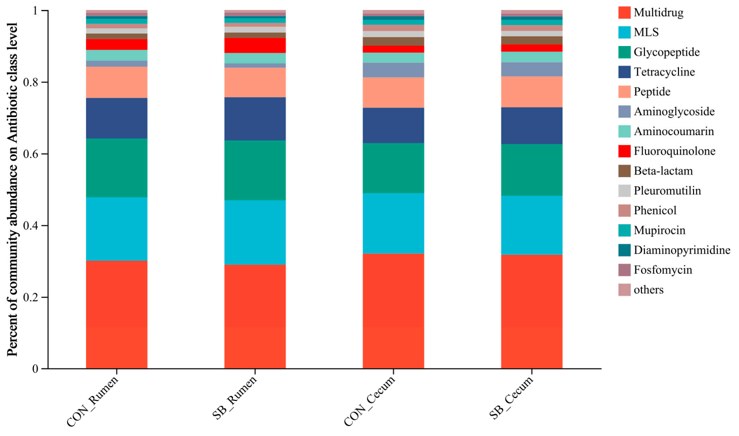

B

Supplementary Figure S3. Composition of VFGs at functional level 2 among the four groups (A). Composition of ARGs at antibiotic class among the four groups (B).
